# Automated synthesis of backbone protected peptides†
†Electronic supplementary information (ESI) available: Synthesis and characterization of peptides, experimental conditions. See DOI: 10.1039/c4cc03065f



**DOI:** 10.1039/c4cc03065f

**Published:** 2014-06-18

**Authors:** Abu-Baker M. Abdel-Aal, George Papageorgiou, Martin Quibell, John Offer

**Affiliations:** a MRC National Institiute for Medical Research , The Ridgeway , London , UK . Email: joffer@nimr.mrc.ac.uk ; Tel: +44 (0)20 8816 2082

## Abstract

The synthesis of peptides rich in aggregation prone sequences can be improved with backbone protection. We report the automated introduction of backbone protection to a peptide. This new method was applied in a fully-automated synthesis, giving improved handling, quality and yield of several challenging target sequences.

## Abstract 2

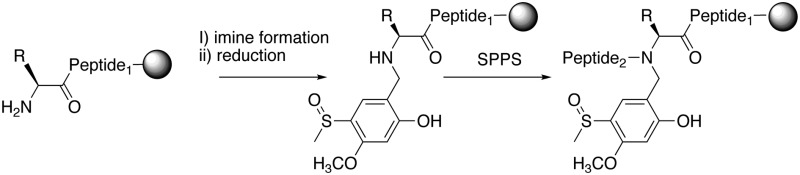
The automated introduction of removable substitution along a peptide backbone prevents chain-association and synthesis failure.

Solid phase peptide synthesis (SPPS) is a powerful technology for the chemical synthesis of peptides and small proteins. However, access to many targets is often complicated and sometimes precluded by the occurrence of so-called difficult sequences.^1,2^ When encountered during SPPS difficult sequences are associated with a collapse of the swollen resin volume, incomplete acylation and in the case of Fmoc/*t*Bu synthesis, incomplete deprotection steps, extending over several residues.^3,4^ As the cause of difficult sequences is intermolecular chain association, double coupling provides no improvement. Pioneering work by Sheppard and co-workers demonstrated that introducing proline into an aggregating peptide sequence, before the onset of aggregation prevented interchain association by removal of hydrogen bonding.^1^ By extension, they also demonstrated that reversible alkylation of the peptide backbone suppressed interchain association and was a general solution to the difficult sequence problem.^5^ Reversible substitution of the amide bond was called ‘backbone protection’ when introduced by Weygand and co-workers because of similarities to the protecting group strategies then in development.^6^ However, it is only rarely necessary to protect the amide bond itself.^7^ Amongst the many backbone protection groups available the most widely used are the commercially available pseudoprolines (ψ-Pro **1** Scheme 1).^8^ Pseudoprolines have revolutionized Fmoc/*t*Bu synthesis by enabling the synthesis of previously intractable peptides, unobtainable even by *in situ* neutralisation Boc protocols.^9^ Their key advantage is that they can be introduced with great convenience as dipeptide building blocks^10^ and have been successfully applied to enable synthesis of difficult sequences^11,12^ and long peptides.^13^ However, their use is unfortunately limited to those sequences containing conveniently positioned X-Ser or X-Thr. Backbone amide protection was first investigated (Dmb **2** Scheme 1) for its dramatic effect on peptide solubility.^6^ The effect of Dmb **2** on improving peptide solubility was explored in detail by Narita and co-workers.^14^ Current opinion considers that the poor solubility observed for many peptide sequences in solution reappears on the solid phase as difficult peptides and backbone protection acts in both cases by disrupting structure formation.^1^ A fully solvated peptide-resin should give the best coupling kinetics and evidence from NMR and I.R. studies on aggregating peptides on solid phase supports this model.^15,16^ Many novel backbone protection strategies have been developed but the increased steric hindrance that accompanies the introduction of a secondary amine into a sequence prohibits quantitative coupling using standard conditions (except with glycine) and has limited the wider adoption of these new backbone protection strategies.^17^ A solution to overcome this obstacle harnessed an intramolecular acyl transfer. This was achieved by the use of 2-hydroxy-4-methoxybenzyl (Hmb) **3** which can be considered as a simple modification of Dmb **2** (Scheme 1). Acylation of the exposed 2-hydroxy position gave a phenyl ester, positioned for acyl migration through a constrained, six-membered ring. This procedure gave quantitative coupling under favourable conditions. However, the kinetics of acyl transfer were slow and variable between residues. Additionally, the optimised non-standard conditions used, consisting of a symmetric anhydride in dichloromethane, were difficult to automate.^5^ Ideally, for practical convenience the coupling onto the secondary amine should be performed under standard conditions. Clearly, with a more reactive internal ester the acylation/migration step could be accelerated. This had previously been achieved by modifying Hmb with an electron withdrawing group *para* to the 2-hydroxy position.^18–20^ Based on these considerations, we chose a variant of the sulfoxide modified Hmb previously reported by us Hmsb **4** (Scheme 2B).^18,20^ The use of sulfoxide, resolves the problem of how to introduce an electron withdrawing group to the backbone protection without making the modification irreversibly stable to acid, as it can be mildly reduced to the acid labile thioether. For a chemical reaction to be generally applicable to the solid phase it has to be quantitative. Ede and co-workers had demonstrated introduction of Hmb to a resin-bound peptide by exploiting the unusually stable Schiff base,^21^ formed between a salicylaldehyde and resin-bound primary amine, stable to washing and subsequently quantitatively reduced on resin.^22^ Therefore a successful demonstration of quantitative on-resin reductive amination of salicylaldehydes, with a group capable of assisting its own acylation under standard conditions would allow automation of backbone protection installation.

**Scheme 1:**
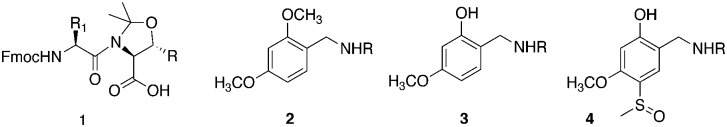
Peptide backbone protection groups. (1) Pseudoproline(ψ-Pro), R = H, Ser; R = CH_3_, Thr. (2) Dimethoxylbenzyl (Dmb); (3) 2-hydroxy-4-methoxybenzyl (Hmb), (4) 2-hydroxy-4-methoxy-5-methylsulfinyl benzyl (Hmsb).

**Scheme 2:**
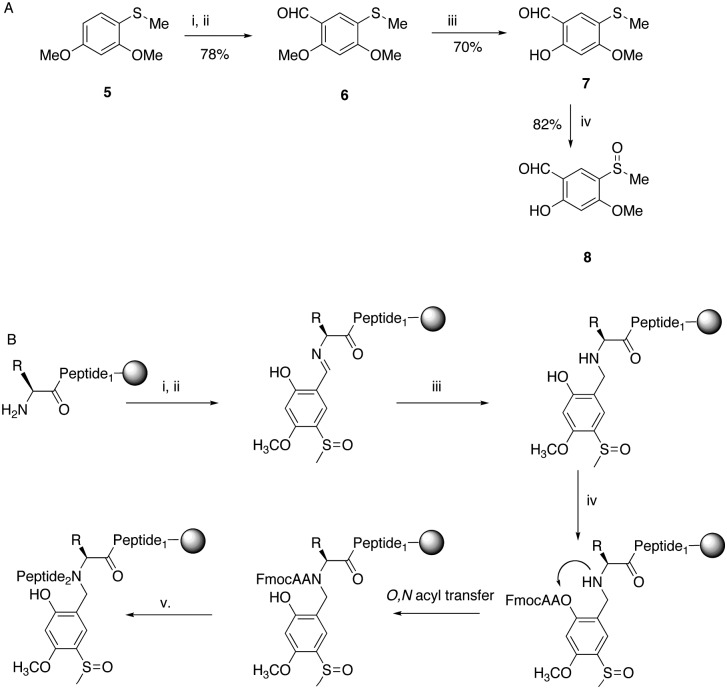
(A) Synthesis of Hmsb backbone protection. (i) DMF, POCl_3_, 0 °C; (ii) ClCH_2_CH_2_Cl, 80 °C; (iii) BBr_3_·SMe_2_, CH_2_Cl_2_, 0 °C; (iv) *m*-CPBA, CHCl_3_, –10 °C. (B) Automated introduction of backbone protection to peptide on solid phase. (i) Imine formation; salicylaldehyde **8** 1.1 equivalent to resin loading; (ii) DMF wash; (iii) NaBH_4_/DMF; (iv) FmocAA, HCTU/DIEA, 30 min; (v) SPPS.

Synthesis of the salicylaldehyde **8** (Scheme 2) was simplified from an earlier route and obtained in a good overall yield from commercial starting materials (ESI†). Chemoselective removal of the methylether by boron tribromide gave salicylaldehyde **7**. For automation to work the sulfoxide group had to be present for imine formation on the solid phase and survive the conditions of reductive amination (Scheme 2B). Previously, we had introduced backbone protection by synthesizing an amino acid building block and oxidizing the thioether to the sulfoxide *after* preparation of the building block.^18^ However, oxidation is difficult to perform quantitatively on-resin and is also not compatible with all amino acids. Therefore thioether **7** was cautiously oxidized to the sulfoxide **8** in the presence of the unprotected aldehyde function. With the sulfoxide substituted salicylaldehyde **8** in hand we first needed to prove that the salicylaldehyde could be installed quantitatively with the sulfoxide intact. In contrast to many aldehydes where imine formation is slow, salicylaldehydes form stable imines rapidly.^21^

Formation of the imine with a single equivalent of **8** gave an intense yellow colouration, the resin was washed to remove excess salicylaldehyde and treated with an additional single equivalent of salicylaldehyde before thoroughly washing with dimethylformamide (DMF). NaBH_4_ in DMF was added and the strong yellow colouration of the Schiff base quickly faded. Analytical HPLC of a test cleavage showed the presence of a single product with the correct mass of the target peptide with backbone protection attached, bearing intact sulfoxide **4** (ESI,† Fig. S1).

This technology was demonstrated by the improved preparation of a ‘difficult sequence’ derived from influenza virus Hemagglutinin, reported by Sampson and co-workers (Fig. 1).^11^ For comparison, the test sequence was synthesised without backbone protection on rink amide resin (Fig. 1A). The peptide aggregated around the tenth residue (Ser^10^) and provided a poor quality crude product with many deletion impurities (Fig. 1A). The use of microwave gave a marginally improved product, however it still contained major deletion sequences (Fig. 1C). In contrast, fully automated addition of Hmsb **4** backbone protection to Ala^9^, followed by a standard coupling cycle for the addition of Lys^8^ provided a greatly improved crude product (Fig. 1(B & D)). The results using conventional automated synthesis and peptide coupling agents *O*-(6-chlorobenzotriazol-1-yl)-*N*,*N*,*N*′,*N*′-tetramethyluronium hexafluorophosphate. (HCTU)/DIEA, 30 min or microwave (DIC/1-hydroxybenzotriazole (HOBt), 10 min) were comparable.

**Fig. 1:**
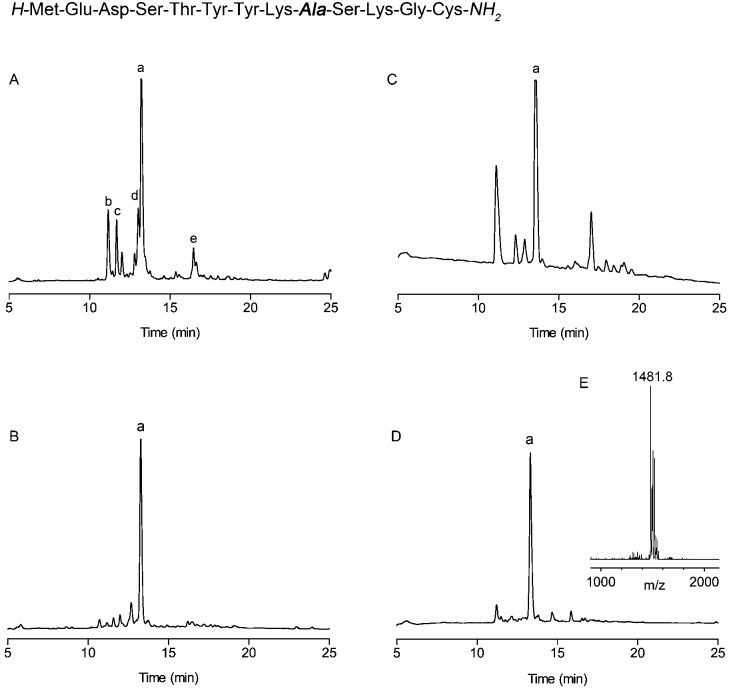
Synthesis of a difficult peptide sequence from influenza virus Hemagglutinin.^11^ Analytical HPLC traces of crude product prepared using: (A) conventional automated SPPS, (a = target peptide, b = deletion of Met^1^-Glu^2^-Asp^3^, c = deletion of Met^1^-Glu^2^, d = deletion of Glu^1^, e = *t*-butylated product). (B) Conventional automated SPPS with backbone protection at Ala^9^. (C) Automated microwave assisted SPPS. (D) Automated microwave-assisted SPPS backbone protection at Ala^9^. (E) MALDI-MS of target peptide, a, (calculated mass [M + H]^+^ = 1481.6 *m*/*z*) peptide cleavage conditions: TFA/TMSBr/thioanisole/ethanedithiol (1.0 : 0.05 : 0.05 : 0.025 v/v), 1.0 h. HPLC conditions: RP-C18, 0–50% (0.1% TFA, 90% CH_3_CN) in 30 min, 1 mL min^–1^.

However, with conventional automated synthesis an additional dichloromethane mix/wash step for 1 h gave a noticeable improvement to the initial product purity, presumably by favouring intramolecular acyl migration.^5,23^ The recovered yield after preparative HPLC was much higher, approximately 30%, for both cases, in contrast to 3% for conventional SPPS and 8% for microwave assisted SPPS, reflecting the less challenging purification task and the practical advantages of using backbone protection. The Hmsb backbone protecting group requires the sulfoxide to be reduced to a thioether for clean deprotection. Previously we used ammonium iodide and dimethylsulfide,^18^ but we found trimethylsilyl bromide (TmsBr) and thioanisole in the cleavage cocktail more convenient.^24^ All the peptides were cleaved using the same cleavage cocktail for comparison of the crude product (Scheme 3). The backbone protection can also be retained on the side-chain deprotected peptide for its beneficial solubility effects.

**Scheme 3:**
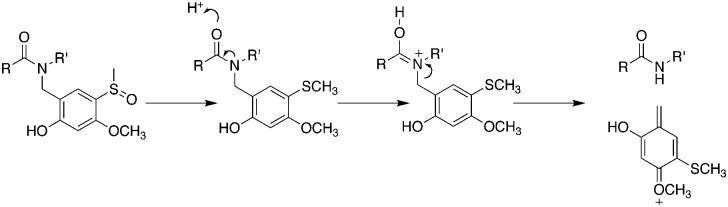
Mechanism of removal of Hmsb backbone protection. TFA/TMSBr/thioanisole/ethanedithiol (1.0 : 0.05 : 0.05 : 0.025 v/v).

In the absence of an *a priori* method to predict an aggregation-prone sequence, a pragmatic approach would be to include a synthetic cycle to protect every sixth residue and prevent any potential aggregation. Polyalanine (with a C-terminal Val) is the prototypical difficult sequence, identified by Merrifield and co-workers and frequently used since as a benchmark.^25–27^ Furthermore, homooligomers of alanine have become the subject of interest because of their biological relevance; as they are one of the most common homopeptide repeats and expanded polyalanine repeats are central to several neurodegenerative diseases.^28^ Aggregation begins at the 5th alanine added from C-terminus and the addition of further alanine residues becomes increasingly troublesome.^26^ The crude product at any of the later stages would be highly insoluble and difficult to analyse or purify. In contrast, automated introduction of two appropriately cited Hmsb backbone protecting groups (at positions Ala^8^ and Ala^14^) using microwave protocols on a Tentagel resin not only prevented aggregation during SPPS, but also by retention on the cleaved crude peptide provided a fully soluble, chemically defined, analogue that is readily analysed and shown to be of high quality with confirmed molecular weight. Circular dichroism of the product gave a spectra characteristic for random coil (Fig. 2C). Cleavage of the Hmsb groups from the polyalanine product removed the solubilising properties afforded by backbone protection, yielding an insoluble, but chemically homogenous product.

**Fig. 2:**
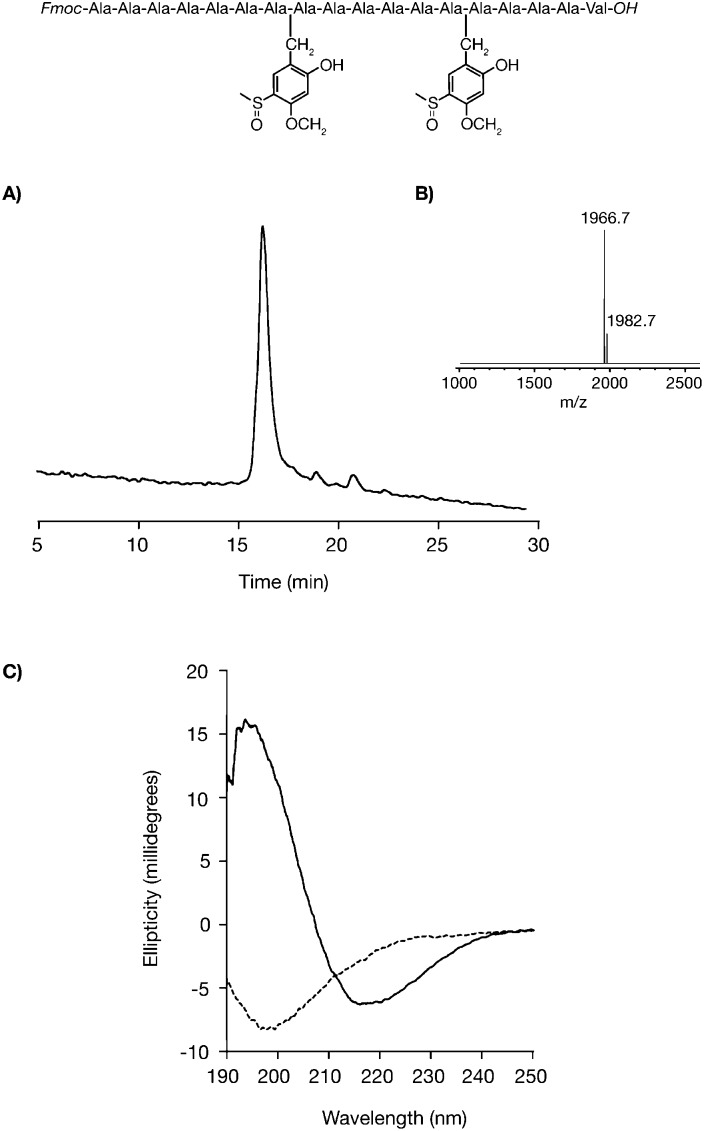
Automated synthesis of the polyalanine octadecapeptide. Fmoc-Ala_7_HmsbAla_6_HmsbAla_4_Val-*OH* (A). Analytical HPLC trace of crude product HPLC conditions: RP-C18, 30–50% B (0.1% TFA, 90% CH_3_CN) in 30 min. 1 mL min^–1^. (B) MALDI-MS of product (calculated mass [M + Na]^+^ = 1965.8 *m*/*z* [M + K]^+^ = 1981.8 *m*/*z*). (C) Circular dichroism of polyalanine containing backbone protection forming random coil (dotted). Aggregated beta-sheets of polyalanine (solid) without backbone protection.

Both previous examples had possessed alanine at the position for backbone protection. We therefore synthesised an additional example, the highly conserved epitope of gp41 that binds tightly to the broadly neutralising 4E10 antibody and previously synthesised with *in situ* neutralisation Boc cycles^29^ We added backbone protection at Leu^679^ using the automated procedure with tryptophan as the following residue. The results demonstrate successful incorporation of backbone protection and improved synthesis (ESI,† Fig. S2).

We have demonstrated that the introduction of backbone protection into a difficult sequence can be fully automated and delivers a significant improvement in yield and quality of previously aggregation prone peptides. The ability to freely add reversible amide protection along the peptide backbone has been a long-term goal for peptide chemists, both to overcome difficult sequences and also to solubilise the peptide in solution. The site of backbone protection is no longer restricted to only two residues. This study has demonstrated the suitability of salicylaldehydes for automated introduction to solid phase by the inclusion of an imine formation/reduction cycle. The Hmbs group actively participates in acylation using an activated acyl transfer so that its reactivity resembles a primary amine more than a secondary amine. With the demonstration of efficient automated introduction, backbone protection can be used preventively at every sixth residue for routine peptide synthesis. The single equivalent of salicylaldehyde required for this approach has potential to be relevant to optimised production of peptides. With the increasing recognition of the importance of peptides as next generation therapeutics and homooligopeptides as interesting clinical materials such a development will meet an urgent need. Of particular interest would be their application to emerging automated flow based techniques for rapid peptide synthesis.^30^ We are currently investigating the scope of this modification, especially its tolerance to β-branched amino acids at the substitution site and its application to longer targets.

This work was supported by a grant-in-aid from the MRC number U117592730.
