# Supplementary material for: Automated synthesis of backbone protected peptides
Source: Chem Commun (Camb). 2014 Jun 18;50(61):8316–9. doi: 10.1039/c4cc03065f (PMC4161149; doi:10.1039/c4cc03065f)
Supplement: Supplementary file 1 [file CC-050-C4CC03065F-s001.pdf]

Electronic Supplementary Material (ESI) for ChemComm.
This journal is © The Royal Society of Chemistry 2014

Electronic Supplementary Information

Automated synthesis of backbone protected peptides

Abu-Baker M. Abdel-Aal, George Papageorgiou, Martin Quibell and John Offer

Abbreviations

CHCA: α-cyano-4-hydroxycinnamic acid. DCM: dichloromethane. DIC: 1,3-diisopropylcarbodiimide. DIEA: N,N-diisopropylethylamine. DMF: N,N-dimethylformamide. Fmoc: 9-fluorenylmethoxycarbonyl. HCTU: *O*-(6-Chlorobenzotriazol-1-yl)-*N*,*N*,*N'*,*N'*-tetramethyluronium hexafluorophosphate. HOBt: 1-hydroxybenzotriazole. MALDI-TOF MS: matrix-assisted laser desorption ionization time-of-flight mass spectrometry. MW: microwave. TFA: trifluoroacetic acid. TFE: 2,2,2-trifluoroethanol.

2,4-Dimethoxy-1-(methylsulfanyl)benzene 5

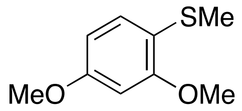

To a solution of 6-hydroxy-1,3-benzoxathiol-2-one (purchased Aldrich CAS 4991-65-5) (16.82 g, 100 mmol) in 2-butanone (250 mL) was added cesium carbonate (73.05 g, 220 mmol) and iodomethane (70.97 g, 500 mmol) and the mixture was refluxed under nitrogen overnight. After cooling to room temperature the solid was filtered off, washed with 2-butanone and the filtrate evaporated. The residue was dissolved in EtOAc (150 mL) and washed with saturated aq. $NaHCO_3$ and brine, dried and evaporated to give **5** (16.05 g, 87%) as white crystals, mp 39-41 °C (Et_2O-hexanes) [lit[1] mp 38-39°C]. ^{1}H NMR (400 MHz, $CDCl_3$): δ 7.18 (d, J = 8.2 Hz, 1 H, ArH), 6.44-6.47 (m, 2 H, ArH), 3.86 (s, 3 H, OCH_3), 3.78 (s, 3 H, OCH_3), 2.36 (s, 3 H, SCH_3).

2,4-Dimethoxy-5-(methylthio)benzaldehyde 6

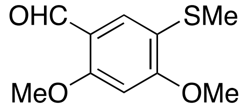

Dry DMF (8.77 g, 120 mmol) was cooled in an ice-bath and treated under nitrogen with freshly distilled phosphorous oxychloride (18.40 g, 120 mmol) and the mixture was stirred at 0-5 ˚C for 30 min. The clear orange solution was treated under nitrogen with a solution of **5** (4.42 g, 24 mmol) in 1,2-dichloroethane (120 mL) and the mixture was first stirred at room temperature for 18 h overnight and then heated to 80 ˚C for further 1.5 h. After cooling to room temperature the solution was diluted with dichloromethane (100 mL), poured into saturated aq. $NaHCO_3$ (300 mL) and stirred vigorously for 30 min. The organic phase was separated and the aq. phase was washed with dichloromethane (2 x 100 mL). The combined organic phases were washed with brine, dried and evaporated to a pale solid. Recrystallisation afforded aldehyde **6** (3.97 g, 78%) as off-white crystals, mp 125°C (EtOAc-hexanes) [lit[2] mp. 124.5-125.5]. ^{1}H NMR (400 MHz, $CDCl_3$): δ 10.27 (s, 1 H, CHO), 7.64 (s 1 H, ArH), 6.41 (s, 1 H, ArH), 3.96 (s, 3 H, OCH_3), 3.92 (s, 3 H, OCH_3), 2.40 (s, 3 H, SCH_3). Anal. Calcd for $C_{10}H_{12}O_3S$: C, 56.59; H, 5.70. Found: C, 56.88; H, 5.67.

2-Hydroxy-4-methoxy-5-(methylthio)benzaldehyde 7

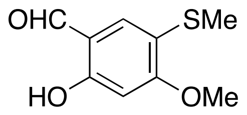

To a solution of **6** (4.25 g, 20 mmol) in dried CH_2Cl_2 (340 mL), cooled to -0 ˚C was added under nitrogen boron tribromide dimethyl sulfide complex (13.13 g, 42 mmol) and the mixture was stirred at 0 ˚C for 4 h. The solution was diluted with CH_2Cl_2 (80 ml) and washed with water, saturated aq. $NaHCO_3$ and brine, dried and evaporated to brown solid. Flash chromatography [EtOAc-hexanes (1:4)] gave salicylaldehyde **7** (1.39 g, 70%) as pale crystals, mp 97-98°C (EtOAc-hexanes). ^{1}H NMR (400 MHz, $CDCl_3$): δ 11.42 (s, 1 H, OH), 9.66 (s, 1 H, CHO), 7.31 (s, 1 H, ArH), 6.38 (s, 1 H, ArH), 3.89 (s, 3 H, OCH_3), 2.35 (s, 3 H, SCH_3). Anal. Calcd for $C_9H_{10}O_3S$: C, 54.53; H, 5.08. Found: C, 54.87; H, 4.95.

2-Hydroxy-4-methoxy-5-(methylsulfinyl)benzaldehyde 8

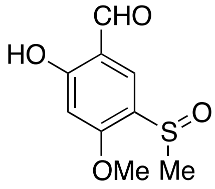

To a solution of salicylaldehyde **7** (4.96 g, 25 mmol) in $CHCl_3$ (350 mL), cooled to -10 °C was added portionwise over 20 min *m*-CPBA (70-75%; 4.31 g, 25 mmol) and the mixture was stirred at -10 °C for 1 h. The solution was concentrated and the residue was flash chromatographed [EtOAc-hexanes (4:1)] to give **8** (4.37 g, 82%) as white crystals, mp 165-167 °C (EtOAc-hexanes). ^{1}H NMR (400 MHz, $CDCl_3$): δ 11.73 (s, 1 H, OH), 9.81 (s, 1 H, CHO), 8.00 (s, 1 H, ArH), 6.47 (s, 1 H, ArH), 3.92 (s, 3 H, OCH_3), 2.75 (s, 3 H, S(O)CH_3). Anal. Calcd for $C_9H_{10}O_4S$: C, 50.46; H, 4.70. Found: C, 50.66; H, 4.65.

Peptide synthesis

Peptides were prepared by standard using a (CEM Liberty1™ Single Channel Microwave Peptide Synthesizer) or (CS Bio 336 automated synthesizer) activation for Fmoc/t-Bu chemistry. Following peptide cleavage, the TFA-cleavage cocktail was filtered, filtrate sparged (nitrogen) and peptides were precipitated with Et_2O (Na-dried, 4.0 °C) and freeze-dried. Peptides were purified by semi-preparative HPLC on a RP-C18 column (22 x 250 mm, Vydac) using linear gradients of CH_3CN in 0.1 % TFA/H_2O with a flow rate of 10 mL.min^{-1}. HPLC gradients were prepared using solvent A (0.1% TFA in H_2O) and solvent B (90 % CH_3CN in 0.1 % TFA). Detection was performed at 214 nm. Peptides were characterized by MALDI-TOF MS on a BRUKER microflex (ion positive linear and reflector mode) using CHCA matrix (10 mg.mL^{-1} in CH_3CN/H_2O/TFA, 50:50:0.1).

Conventional automated protocol

Peptides were prepared using HCTU/DIEA (CS Bio 336 automated synthesizer) activation for Fmoc/t-Bu chemistry. Fmoc-deprotection was performed using 20% piperidine in DMF in two stages with an initial 3.0-min followed by 7.0-min-deprotection. All coupling reactions were performed with 5-fold excess Fmoc-Amino acid-OH/HCTU/DIEA for 30 min. The backbone protecting group was introduced in two steps. The salicylaldehyde **8** dissolved in DMF was added to peptide-resin (two 30-min-cycles x 1.0 eq., 0.01 M) followed by DMF wash cycle, this gave a strong yellow colour to the resin indicating imine formation. Reduction with $NaBH_4$ dissolved in DMF (filtered, PVDF 0.2 µm) (two 15-min-cycles x 5.0 eq., 0.1 M) followed by thorough DMF wash. The following amino acid following insertion of the

backbone protecting group was coupled to the alkylated amino group as previously followed by DCM wash, 1.0 h shaking in DCM, followed by DMF wash, and 30 min shaking in DMF.

MW-assisted automated protocol

Peptides were prepared using DIC/HOBt (CEM Liberty1™ Single Channel Microwave Peptide Synthesizer) activation for Fmoc/t-Bu chemistry. Fmoc-deprotection was performed using 20% piperidine in DMF in two stages with an initial 0.5-min followed by 3.0-min-deprotection (7.0 mL, 40W, 75 °C). Coupling reactions were performed with 5-fold excess Fmoc-Amino acid-OH/DIC/HOBt (1.0: 0.8: 2.0) for 10 min, 25 W, 75 °C. The backbone-protecting group was inserted in two-step reductive alkylation. First, the salicylaldehyde **8** was added to the peptide resin (two 10-min-cycles x1.0 eq., 0.02 M, 25W, 50°C) followed by thorough DMF wash. Second, reduction was performed by treatment with a solution of $NaBH_4$ in DMF (two 15-min-cycles x 5.0 eq., 0.1 M, rt) followed by thorough DMF wash. Amino acid following insertion of the backbone protecting group was coupled directly to alkylated amino group with 5-fold excess Fmoc-Amino acid-OH/DIC/HOBt (1.0: 0.8: 1.0) for 30 min, 25 W, 75 °C.

Synthesis of *H*-Met-Glu-Asp-Ser-Thr-Tyr-Tyr-Lys-Ala-Ser-Lys-Gly-Cys-*NH*$_2$

Method 1: Standard SPPS (No backbone protection). The peptide was assembled on Rink amide resin (Polymer labs) (250 mg, resin loading 0.39 mmol/g) using standard HCTU/DIEA activation for Fmoc/t-Bu chemistry (CS Bio 336 automated synthesizer). Couplings were carried out with a 10-fold excess of activated amino acid for a minimum of 30 min. Synthesis yielded 431 mg of dried peptide-resin. A portion of the peptide-resin (200 mg) was cleaved using a mixture of TFA/ TMSBr/ thioanisole/ EDT (1.0:0.05:0.05:0.025 v/v, 1 mL, 1.0 h). HPLC purification (0-50% B in 30 min) yielded 2.1 mg of peptide, 3.0% yield (purity > 95%).

Method 2: Microwave-assisted SPPS (No backbone protection). The peptide was assembled on Rink amide resin (Polymer labs) (250 mg, resin loading 0.39 mmol/g) using standard DIC/HOBt activation for Fmoc/t-Bu chemistry (CEM Liberty1™ Single Channel Microwave Peptide Synthesizer). Couplings were carried out with a 5-fold excess of activated amino acid. Synthesis yielded 400 mg of dried peptide-resin. A portion of the peptide-resin (70.0 mg) was cleaved using a mixture of TFA/ TMSBr/ thioanisole/ EDT (1.0:0.05:0.05:0.025 v/v, 1 mL, 1.0 h). HPLC purification (0-50% B in 30 min) yielded 1.9 mg of peptide, 7.6% yield (purity > 98%).

Method 3: New room temperature automated protocol. The peptide was assembled on Rink amide resin (Polymer labs) (125 mg, resin loading 0.39 mmol/g) using HCTU/DIEA activation for Fmoc/t-Bu chemistry (CS Bio 336 automated synthesizer). Couplings were carried out with a 10-fold excess of activated amino acid for a minimum of 30 min. The backbone-protecting group **8** was introduced at Ala^9 followed by direct coupling of Lys^8 as described in the general rt automated protocol. Synthesis yielded 232.0 mg of dried peptide-resin. A portion of the peptide-resin (40 mg) was cleaved using a mixture of TFA/ TMSBr/ thioanisole/ EDT (1.0:0.05:0.05:0.025 v/v, 1.0 mL, 1.0 h). HPLC purification (0-50% B in 30 min) yielded 3.7 mg of peptide, 28.8 % yield (purity > 98%).

Method 4: New MW-assisted automated protocol. The peptide was assembled on Rink amide resin (Polymer labs) (250 mg, resin loading 0.39 mmol/g) using DIC/HOBt activation for Fmoc/t-Bu chemistry (CEM Liberty1™ Single Channel Microwave Peptide Synthesizer). Couplings were carried out with a 5-fold excess of activated amino acid. Backbone-protection **8** was inserted after Fmoc deprotection of Ala^9 followed by direct coupling of Lys^8 as described in the general microwave-assisted protocol. Synthesis yielded 432 mg of dried peptide-resin. A portion of the peptide-resin (50 mg) was cleaved using a mixture of TFA/ TMSBr/ thioanisole/ EDT (1.0:0.05:0.05:0.025 v/v, 1mL, 1.0 h). HPLC purification (0-50% B in 30 min) yielded 5.8 mg of peptide, 33.8% yield (purity > 98%).

Synthesis of *Fmoc*-Ala$_7$-(Hmsb)Ala-Ala$_5$-(Hmsb)Ala-Ala$_3$-Val-*OH*

The peptide was assembled on Fmoc-Val-NovaSyn® TGT resin (Novabiochem®) (227 mg, resin loading 0.22 mmol/g) using DIC/HOBt activation for Fmoc/t-Bu chemistry (CEM Liberty1™ Single Channel Microwave Peptide Synthesizer) as described in the general microwave-assisted protocol. Couplings were carried out with a 5-fold excess of activated amino acid. Two backbone protecting groups were inserted after Fmoc-deprotection of Ala^{8} and Ala^{14}. Test cleavage using TFE/DCM (1:1 v/v, 1.0 mL, 1.5 h) was performed after insertion of the first backbone protecing group to check completion of the insertion **(Figure S1)**. Synthesis yielded 270 mg of dried peptide-resin. A portion of the peptide-resin (50 mg) was cleaved using TFE/DCM (1:1 v/v, 1.0 mL, 1.5 h) and HPLC purification (0-50% B in 30 min) yielded 5.7 mg of peptide, 31.6 % yield (purity > 98 %). m/z = 1966.7 $[M+Na]^{+}$ (calc.: 1965.8), m/z = 1982.7 $[M+K]^{+}$ (calc.: 1981.8).

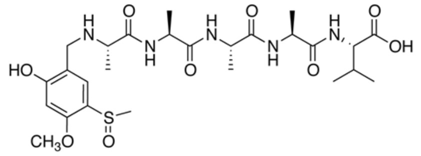

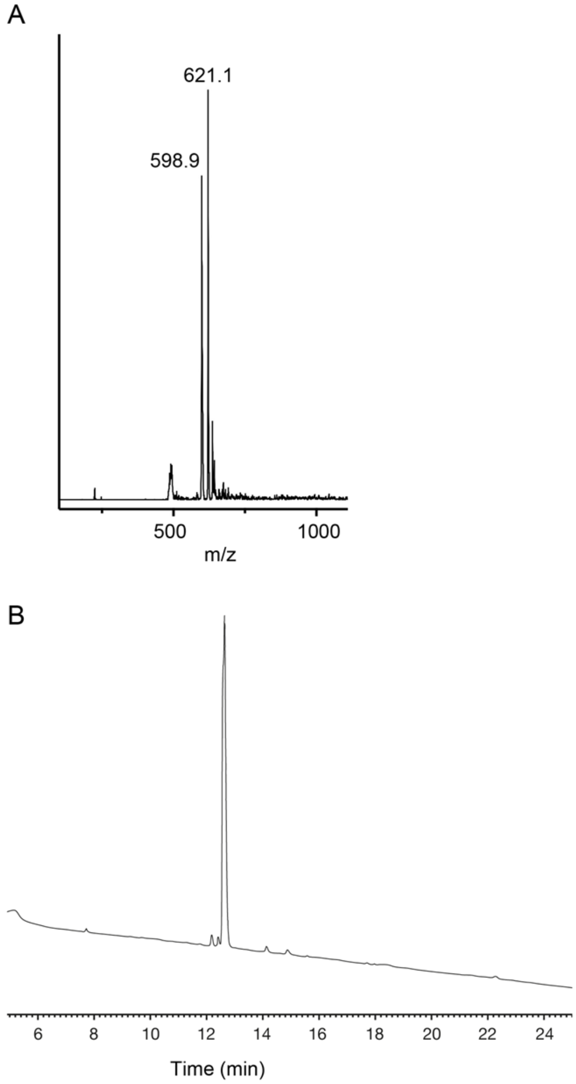

Figure S1: Insertion of Hmsb backbone protecting group. A) MALDI-MS of product. Calculated mass $[M+H]^{+}$= 600.2 m/z $[M+Na]^{+}$= 622.2 m/z. B) analytical HPLC traces of crude product using RP-C18, 10-50% CH_3CN in 0.1% TFA over 30 min, 1 mL.min^{-1}.

Synthesis of gp41 Glu^{662}-Lys^{683} MPER peptide

The peptide was assembled on Rink amide resin (Polymer labs) (250 mg, resin loading 0.39 mmol/g) using DIC/HOBt activation for Fmoc/t-Bu chemistry (CEM Liberty1™ Single Channel Microwave Peptide Synthesizer). Couplings were carried out with a 5-fold excess of activated amino acid. The backbone protecting group was inserted after Fmoc deprotection of Leu^{679} followed by direct coupling of Trp^{678} as described in the general microwave-assisted protocol. Synthesis yielded 595 mg of dried peptide-resin. A portion of the peptide-resin (100 mg) was cleaved using a mixture of TFA/ TMSBr/ thioanisole/ EDT (1.0:0.05:0.05:0.025 v/v, 1 mL, 1.0 h). HPLC purification (35-55% B in 30 min) yielded 9.0 mg of peptide, 18.3 % yield (purity > 98%). Analytical HPLC conditions were run on RP-C18, 40-60% CH_3CN in 0.1% TFA over 30 min, 1 $mL.min^{-1}$. MALDI-MS of the product: m/z= 2925.2 (calculated mass $[M+H]^+$= 2925.5 m/z), **Figure S2**.

H-Glu-Leu-Asp-Lys-Trp-Ala-Ser-Leu-Trp-Asn-Trp-Phe-Asn-Ile-Thr-Asn-Trp-***Leu***-Trp-Tyr-Ile-Lys-NH_2

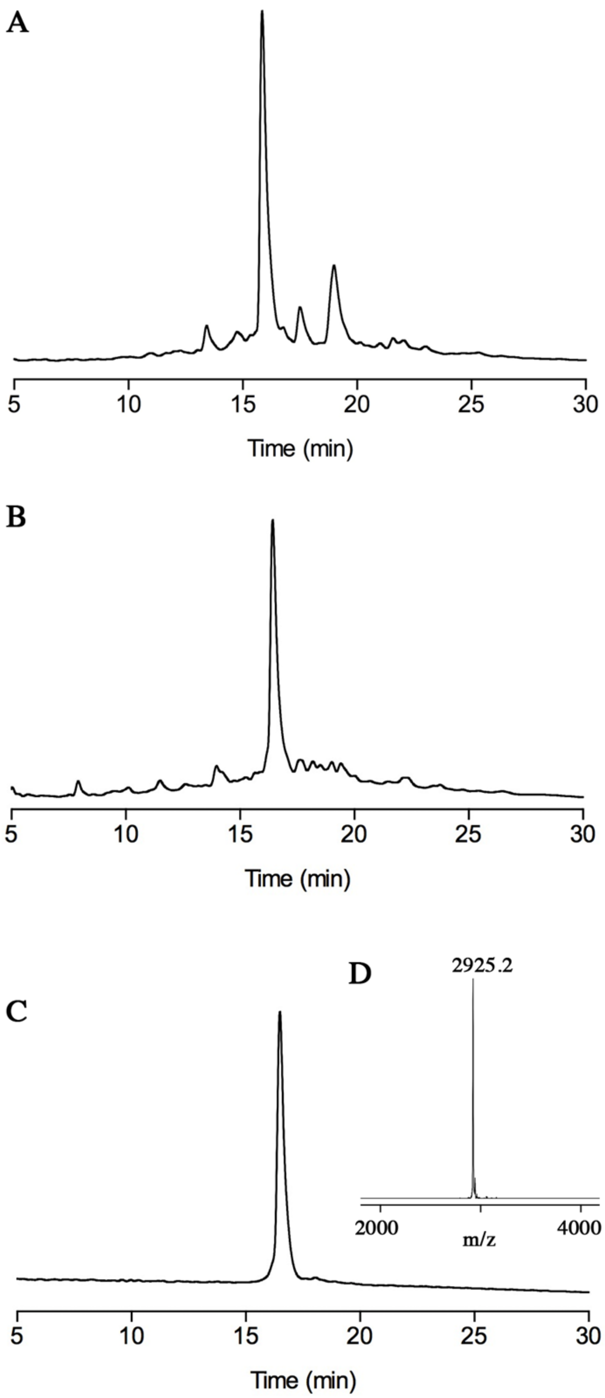

Figure S2: Synthesis of gp41 Glu^{662}-Lys^{683} MPER peptide. A) Analytical HPLC traces of crude product synthesized using microwave-assisted SPPS, B) Analytical HPLC traces of crude product synthesized using new microwave-assisted automated protocol with backbone protecting group inserted at the amino group of Leu^{679}, C) Analytical HPLC traces of pure product. D) MALDI-MS of HPLC-purified product (calculated mass $[M+H]^+$= 2925.5 m/z). Peptide cleavage mixture: TFA/TMSBr/thioanisole/EDT (1.0:0.05:0.05:0.025 v/v), 1.0 h. HPLC conditions: RP-C18, 40-60% CH_3CN in 0.1% TFA over 30 min, 1 $mL.min^{-1}$.

1. C. M. Suter and H. L. Hansen, *J. Am. Chem. Soc.,*1933, **55**, 2080-2082.
2. P. Jacob, III, G. Anderson, III, C. K. Meshul, A. T. Shulgin and N. Castagnoli, Jr., *J. Med. Chemistry*, 1977, **20**, 1235-1239.
